# Deep Neural Network-Driven Reliability Assessment Methodology for Total Ionizing Dose Effects in FDSOI MOSFETs

**DOI:** 10.3390/mi17101154

**Published:** 2026-09-30

**Authors:** Hanying Deng, Yu Wang, Jinshun Bi, Jianbin Jiao, Yue Ma, Baihong Chen, Yundong Xuan

**Affiliations:** 1 Department of Circuits, School of Emergency Management Science and Engineering, University of Chinese Academy of Sciences, Beijing 100049, China; denghanying23@mails.ucas.ac.cn (H.D.); jiaojb@ucas.ac.cn (J.J.); 2 School of Integrated Circuits, Guizhou Normal University, Guiyang 550025, China; likewy1234@gmail.com; 3 University of Chinese Academy of Sciences, Beijing 100049, China; 4 Institute of Microelectronics, Chinese Academy of Sciences, Beijing 100029, China; mayue@ime.ac.cn (Y.M.); chenbaihong@ime.ac.cn (B.C.); 5 School of Integrated Circuits, University of Chinese Academy of Sciences, Beijing 100049, China

**Keywords:** total ionizing dose (TID), FDSOI MOSFET, technology computer-aided design (TCAD), neural networks

## Abstract

This work investigates the effects of total ionizing dose (TID) on 22 nm FDSOI MOSFET devices through combined technology computer-aided design (TCAD) simulations and actual TID irradiation experiments, establishing a comprehensive dataset of radiation-induced degradation characteristics. By utilizing both simulated and experimental radiation data as training foundations, this research developed and optimized a deep neural network (DNN) capable of conducting efficient and accurate reliability analysis of TID effects under various radiation conditions, with demonstrated effectiveness in predicting device performance degradation across different TID ranges. The methodology integrates physical simulations with deep learning approaches, where TCAD simulations and irradiation experiments capture fundamental device physics under radiation exposure, while the DNN enables rapid performance prediction. The resulting framework achieves superior prediction accuracy while significantly reducing computational costs compared to conventional simulation methods, providing a powerful solution for evaluating electronic device reliability in complex radiation environments.

## 1. Introduction

With the rapid advancement of semiconductor fabrication processes, the continuous scaling of device critical dimensions (CD) has led to increasingly pronounced adverse effects. Recent studies on emerging semiconductor and optoelectronic devices have also emphasized that carrier dynamics, structural engineering, stability, and degradation behavior are critical to achieving both high device performance and long-term reliability [1,2]. In this context, Silicon-on-Insulator (SOI) technology has emerged as a prominent solution due to its unique advantages [3].

SOI MOSFETs employ a buried oxide (BOX) layer to fully isolate the silicon channel from the substrate, effectively suppressing short-channel effects (SCEs) such as threshold voltage shift and Drain-Induced Barrier Lowering (DIBL) caused by channel scaling [4]. Partially Depleted SOI (PDSOI) was among the earliest commercialized SOI technologies; however, its relatively thick (~100 nm) silicon channel prevents full depletion, leading to threshold voltage roll-off and degraded RF performance [5,6,7,8]. To address these limitations, Fully Depleted SOI (FDSOI) technology was developed [9]. FDSOI reduces the channel thickness to a few nanometers, enabling complete depletion and enhancing gate control while utilizing intrinsic doping to improve carrier mobility. Additionally, the BOX layer minimizes unwanted current leakage, significantly reducing power consumption.

Since the introduction of the first 28 nm FDSOI devices in 2012, their CD have scaled from the 22 nm node down to 14 nm, achieving a remarkable cutoff frequency (fT) of 375 GHz [10,11,12]. FDSOI technology demonstrates superior resilience to space Single-Event Effects (SEE). The BOX layer effectively isolates the channel from the substrate, confining the diffusion of SEE-generated electron-hole pairs (EHPs) in the substrate toward source/drain regions [13], making it particularly suitable for space applications.

However, the relatively large BOX volume renders FDSOI devices more susceptible to Total Ionizing Dose (TID) effects when exposed to gamma or X-ray irradiation [14,15]. In SOI devices, dielectric layers (including gate oxides, BOX, and shallow trench isolation) serve as primary sites for TID-induced degradation [16]. When X-rays or gamma rays interact with these dielectrics, ionization generates EHPs. Due to their higher mobility, electrons are rapidly swept out of the dielectric layer, while holes drift toward dielectric interfaces under electric field modulation and may become trapped at hole trap sites, forming fixed positive charges [17]. With the accumulation of TID, the increasing fixed charge density progressively affects channel turn-on characteristics, reduces threshold voltage, significantly increases subthreshold leakage current, and degrades device reliability significantly.

Recent experimental studies have further clarified the radiation response of advanced 22 nm FDSOI technologies. Yin et al. [18] investigated TID and single-event responses of 22 nm UTBB FDSOI transistors and SRAMs using irradiation experiments, showing clear threshold-voltage degradation and bias-dependent TID behavior. Zhang et al. [19] extended the characterization of 22 nm UTBB FDSOI MOSFETs to doses as high as 100 Mrad(Si), demonstrating that trapped charge in the buried oxide dominates the high-dose degradation and identifying radiation-induced DIBL effects. Termo et al. [20] also reported X-ray characterization of 22 nm FDSOI MOSFETs under ultra-high TID levels and different back-gate biases, confirming the dominant role of BOX charge trapping.

In parallel, TCAD has been extensively used to interpret radiation-induced degradation mechanisms. Recent studies have reproduced TID-induced electrical degradation through radiation-generation and trapped-charge models and have used TCAD to investigate the roles of BOX charge accumulation, bias conditions, and parasitic electrostatic effects. For example, recent TCAD-assisted studies of advanced FDSOI and FinFET technologies have quantitatively linked trapped oxide charge to threshold-voltage and leakage-current degradation. However, full radiation-physics simulations remain computationally expensive when dense dose/bias parameter sweeps are required.

Data-driven surrogate modeling has recently been introduced into radiation-effect analysis to reduce the computational burden of TCAD. Zhao et al. developed a DNN-based approach for predicting single-event responses of FDSOI devices and demonstrated that neural networks can reproduce key transient characteristics at substantially lower computational cost than direct TCAD simulation [21]. Nevertheless, existing learning-based studies have mainly focused on single-event effects, while the integration of experimentally calibrated TID measurements, spatial BOX-charge prediction, and transfer-characteristic reconstruction remains much less explored.

Recent advances in semiconductor device modeling have further highlighted the importance of balancing physical fidelity with computational efficiency, as demonstrated by physics-informed analytical models developed for emerging power semiconductor devices [22].

A closely related recent study by Huang et al. investigated TID and SET effects in 22 nm FDSOI devices using fixed-charge and gamma-radiation TCAD models, with particular emphasis on radiation-induced electrical degradation, back-gate bias effects, and TID–SET coupling [23].

Despite these advances, three limitations remain. First, recent 22 nm FDSOI TID studies have primarily focused on experimental characterization or physics-based TCAD analysis, with limited integration between measured irradiation data and data-driven surrogate modeling. Second, although fixed-charge models can significantly reduce TCAD computational cost, a spatially uniform or empirically prescribed charge distribution cannot fully reproduce the bias-dependent nonuniform hole distribution in the BOX. Third, existing deep-learning studies of FDSOI radiation effects have mainly addressed single-event phenomena, and a physics-informed framework linking TID conditions, spatial BOX charge distributions, TCAD electrical characteristics, and experimentally calibrated device responses has not been sufficiently investigated.

To address these gaps, this work develops an integrated experimental–TCAD–DNN methodology for 22 nm FDSOI nMOSFETs. The experimentally measured TID characteristics are first used to calibrate and validate the TCAD model within the measured dose range. A region-resolved BOX charge representation is then introduced to accelerate the generation of TID degradation data while preserving the bias-dependent spatial charge distribution. Finally, a two-stage DNN framework is constructed to predict the BOX charge distribution and the corresponding electrical characteristics over a broader range of irradiation conditions.

## 2. Analysis of Total Ionizing Dose Effects in FDSOI Devices: Experimental Results

**Figure 1:**
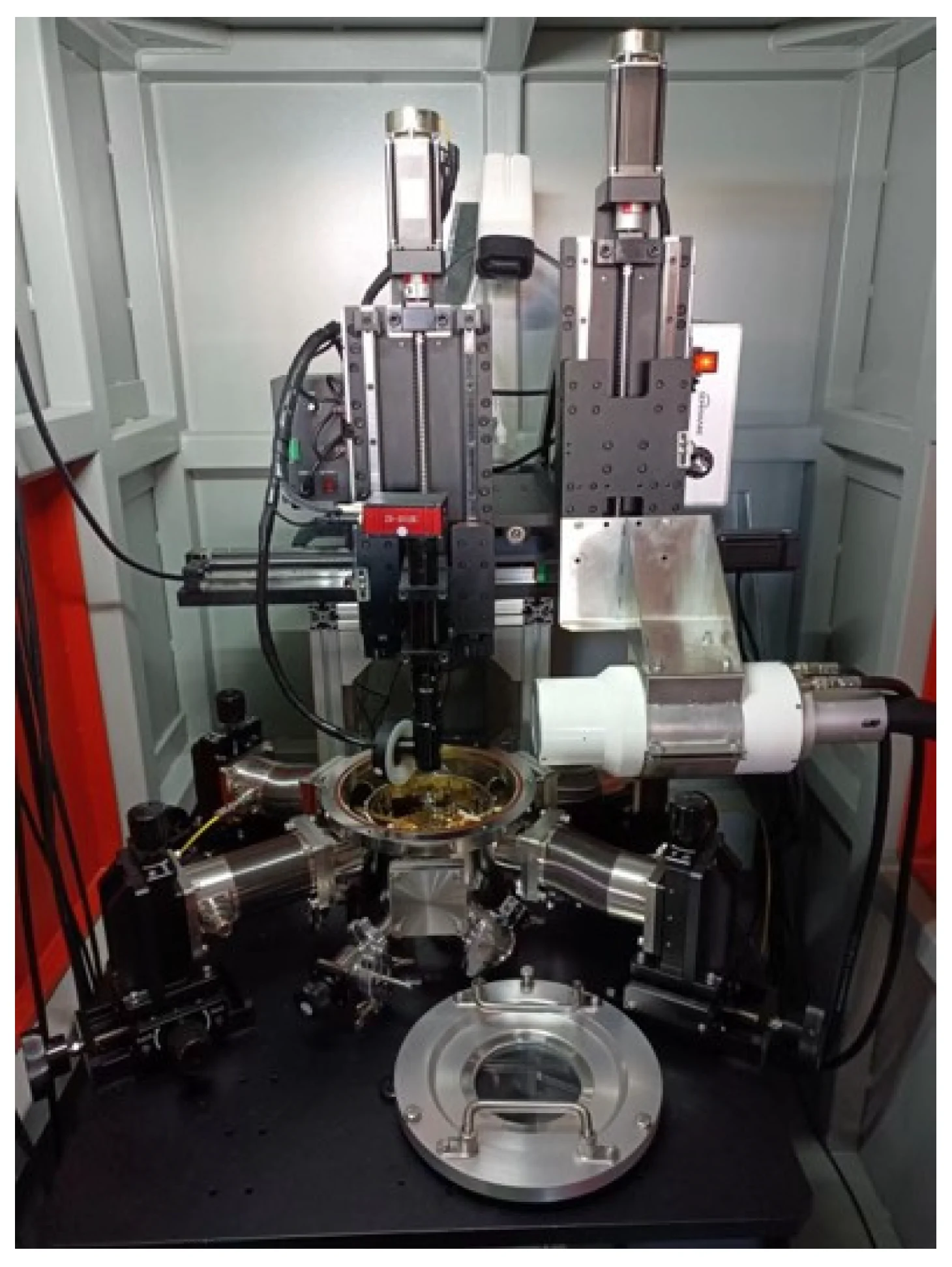
X-ray in-situ simulation total dose irradiation experiment.

**Figure 2:**
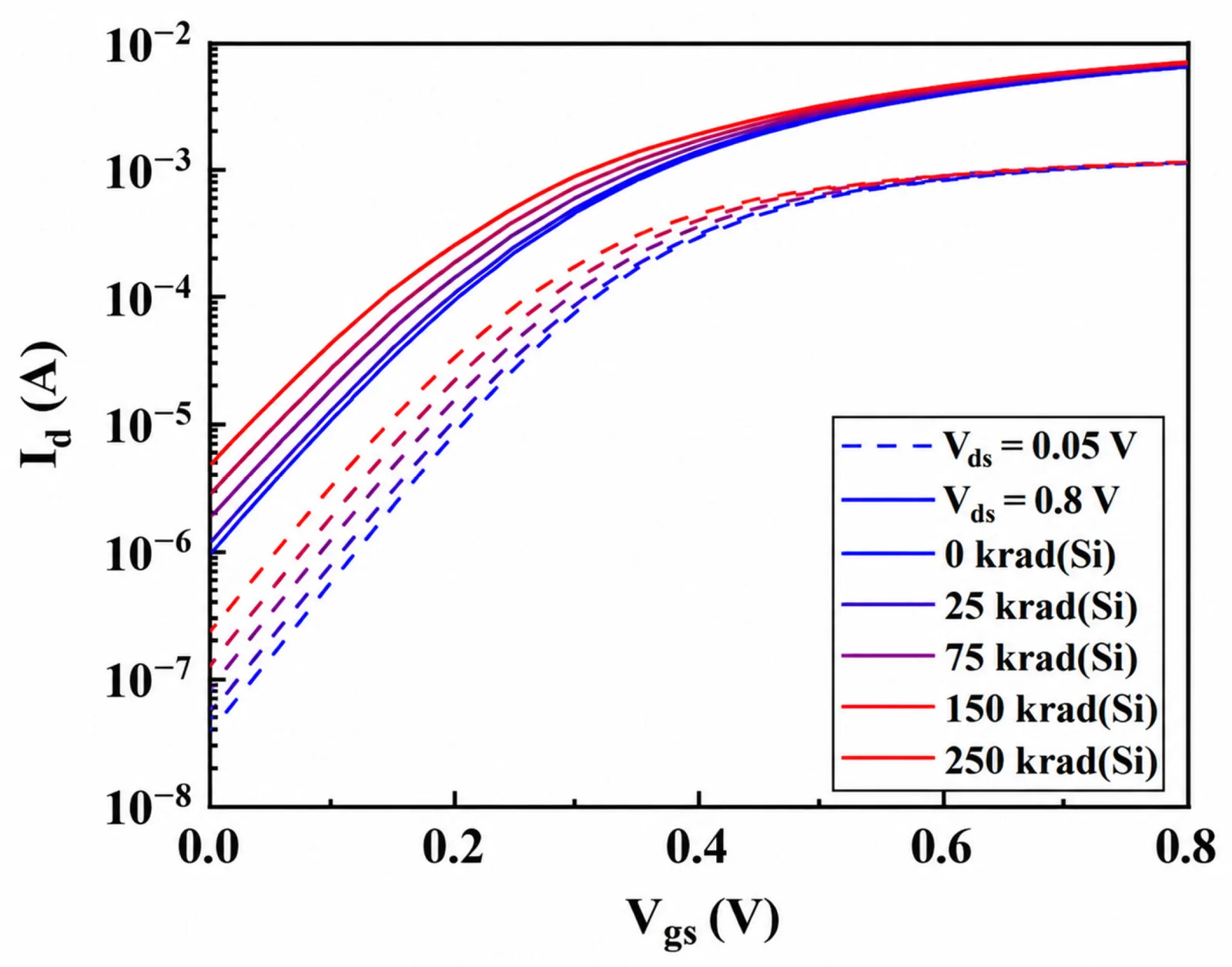
Evolution of the FDSOI MOSFET transfer characteristics under X-ray total-dose irradiation.

A powered on-wafer X-ray irradiation experiment was performed on a 22 nm-node FDSOI nMOSFET to provide direct evidence of radiation-induced electrical degradation and to supply measurement data for TCAD calibration and validation. The device under test was a 22 nm FDSOI nMOSFET based on the GlobalFoundries 22FDX technology [24]. The X-ray source delivered a dose rate of 166.7 rad(Si) s^−1^, and the total dose was increased stepwise from 0 to 250 krad(Si). The same device was electrically characterized after each irradiation step, allowing the evolution of device characteristics to be tracked over the accumulated ionizing dose range. The irradiation setup is shown in Figure 1. Therefore, the transfer characteristics and electrical parameters reported in Figure 2 and Table 1 correspond to measurements from this single device, rather than averaged results obtained from multiple devices.

Figure 2 shows the measured transfer characteristics at different total doses. As the dose increases from 0 to 250 krad(Si), the transfer curves shift toward more negative gate voltage under both Vds = 0.05 V and 0.8 V. At a fixed gate voltage, the drain current increases with dose, indicating that a smaller gate voltage is required to turn on the channel and that the off-state control progressively degrades.

To quantify the electrical degradation, the on-state current (Ion), off-state current (Ioff), on/off current ratio, and threshold voltage (Vth) were extracted from the transfer curves. The results are summarized in Table 1.

When the total dose increases from 0 to 250 krad(Si), Vth decreases from 0.288 to 0.233 V, corresponding to a cumulative negative shift of 55 mV and a relative change of approximately 19.1%. The continuous reduction in Vth is consistent with radiation-induced positive charge accumulated in the BOX and neighboring dielectrics acting as an effective positive back-gate bias, so that the channel turns on at a lower front-gate voltage.

Ion increases from 814 to 883 μA μm^−1^, an absolute increase of 69 μA μm^−1^ or approximately 8.48%. In contrast, Ioff increases from 120 to 582 nA μm^−1^, an absolute increase of 462 nA μm^−1^ or 385%. The much larger relative increase in Ioff shows that TID has a stronger influence on the subthreshold and off-state characteristics than on the on-state current. Overall, the measurements reveal a negative Vth shift, a pronounced increase in off-state leakage, and a decrease in the on/off ratio, providing the experimental foundation for the TID models developed below.

## 3. Total Ionizing Dose Effects in FDSOI Devices: TCAD Simulation and Analysis

TCAD simulations were performed using Synopsys Sentaurus TCAD, version S-2021.06 (Synopsys, Inc., Sunnyvale, CA, USA), on a Linux platform. A two-dimensional FDSOI nMOSFET model was constructed using Sentaurus Structure Editor (SDE), version S-2021.06, and solved using Sentaurus Device (SDevice), version S-2021.06. A nonuniform mesh strategy was adopted, with local refinement at the gate-dielectric/channel interface, BOX/top-silicon interface, and source/drain-to-channel transition regions. The local mesh spacing in the critical regions was reduced to less than 1/50 of the global mesh scale, with additional refinement applied in the BOX and channel regions.

The TCAD device model contains the source, channel, drain, gate dielectric, gate electrode, BOX, back-gate modulation layer, substrate, and substrate contact. For simplified electrical characterization, the channel length is taken to be equal to the gate length. The gate dielectric is represented by a bilayer stack consisting of HfO_2_ with thickness TOX1 and SiO_2_ with thickness TOX2. Because the ultrathin silicon channel (Tchannel = 7 nm) exhibits relatively high series resistance, the raised source/drain thickness is set to TSD = 15 nm to reduce parasitic resistance [25]. Model calibration emphasizes the sensitive parameters NSD, NUB, NSUB, TOX1, TOX2, resist, and areafactor and uses the transfer characteristics of three unirradiated 22 nm FDSOI nMOSFET samples. The calibration targets the subthreshold swing, on-state current, and threshold voltage. The key model parameters are listed in Table 2.

Under TID irradiation, EHPs are generated in the BOX. The more mobile electrons are rapidly swept out, whereas holes migrate toward the BOX/top-silicon interface and may be trapped, forming fixed positive charge centers. Consequently, the spatial electric-field distribution during irradiation strongly influences the post-irradiation device state. Three representative irradiation biases, TG, ON, and OFF, were considered as listed in Table 3. The evolution of the transfer curves and the hole-concentration distribution along the C1 line near the BOX/top-silicon interface were analyzed as a function of irradiation time.

**Figure 3:**
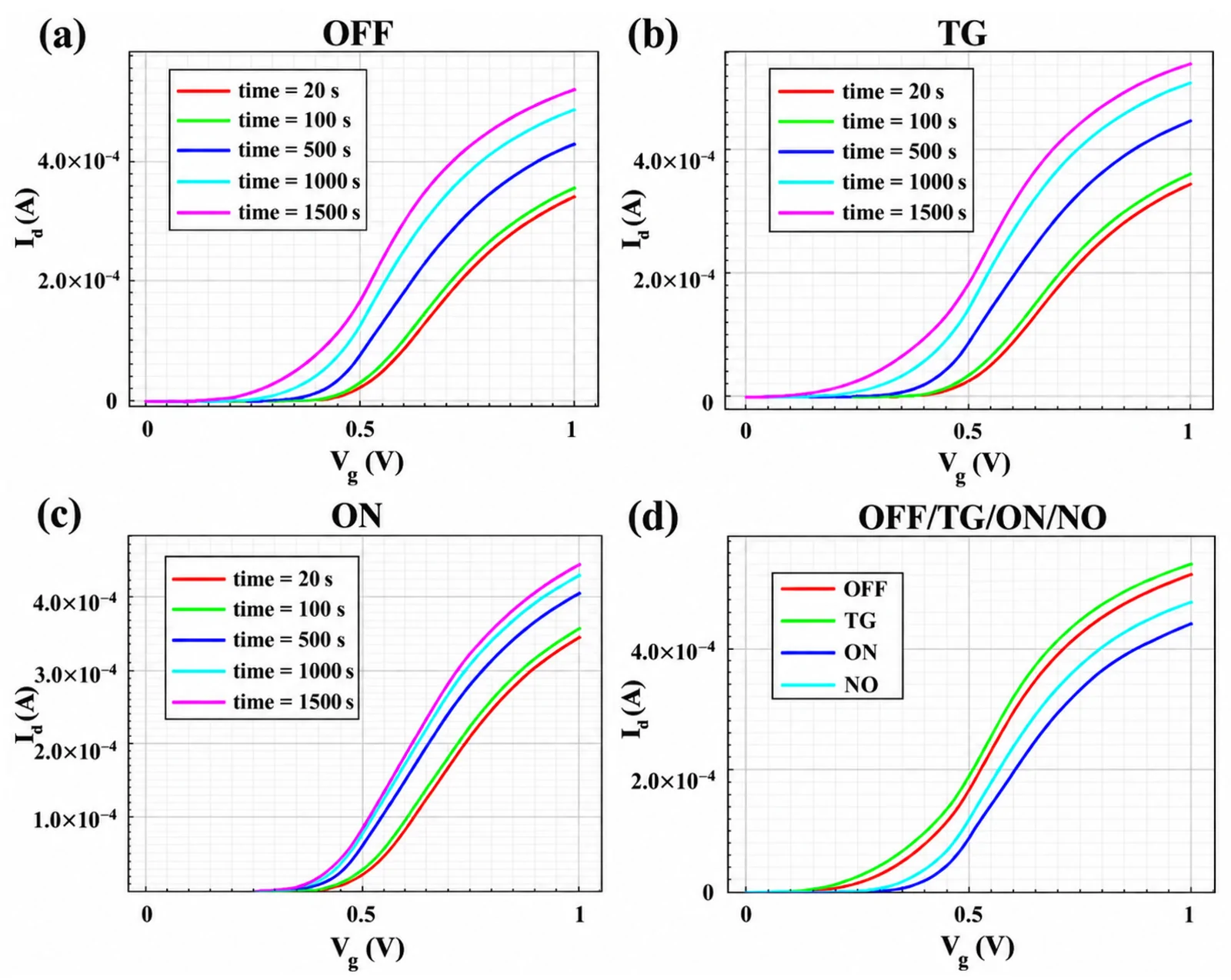
Transfer characteristics as a function of irradiation time under (**a**) OFF bias (Vd = 0.7 V), (**b**) TG bias (Vd = 0.7 V), and (**c**) ON bias (Vd = 0 V), together with (**d**) a comparison among OFF, TG, ON, and NO-bias conditions.

**Figure 4:**
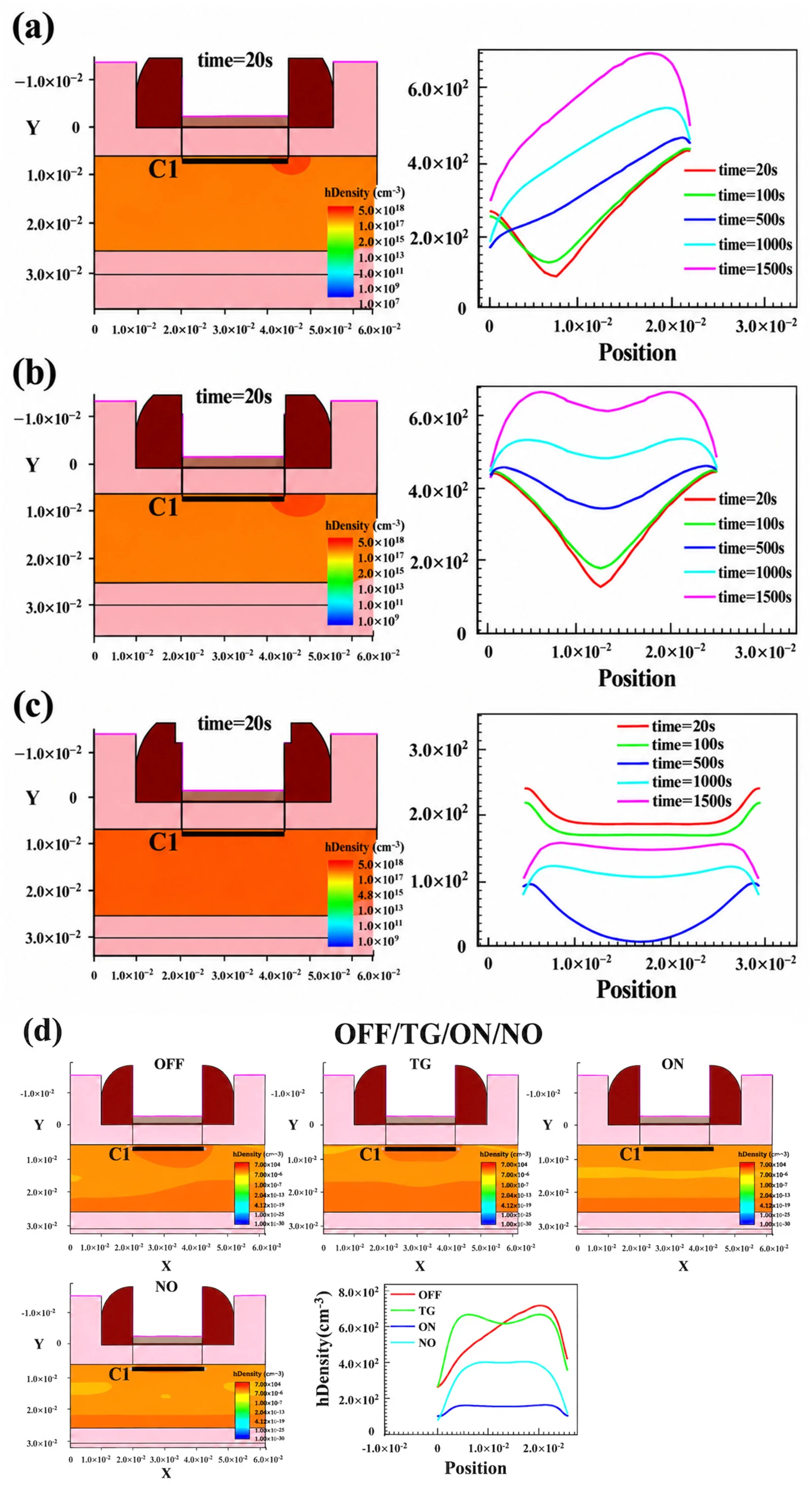
Fixed-hole concentration distributions in the buried oxide (BOX): (**a**) OFF bias, (**b**) TG bias, and (**c**) ON bias at an irradiation time of 20 s; (**d**) comparison of the four bias conditions.

For OFF bias during irradiation from 1 to 1500 s (0–250 krad(Si)), the transfer characteristics progressively shift in the negative direction and the channel turns on earlier, as shown in Figure 3a. The C1-interface hole distribution in Figure 4a shifts toward one side with increasing dose, eventually producing a drain-to-source concentration asymmetry of approximately 7:3. This behavior arises from the lateral electric field established by the drain potential (Vd = 0.7 V), which drives hole transport along the field gradient.

Under TG bias over the same irradiation interval, Figure 3b shows a progressive negative shift in the transfer curves. Meanwhile, the C1 hole distribution in Figure 4b evolves from a double-peaked profile toward a single central peak. The source/drain bias configuration produces a potential well that confines holes near the center; after approximately 300 s, trap-limited transport becomes dominant and the trap occupancy ultimately exceeds 90%.

Under ON bias, the transfer characteristics again shift negatively with increasing dose (Figure 3c), whereas Figure 4c shows a more complex dose-dependent redistribution of holes near the BOX/channel interface. When only the irradiation bias is changed and all other model parameters are held constant, the transfer characteristics shift negatively for OFF, TG, ON, and NO-bias conditions (Figure 3d). The largest shift is observed under TG bias and the smallest under ON bias; the OFF-bias response lies between the TG and NO-bias cases.

The hole-concentration profiles extracted along the C1 line near the BOX/top-silicon interface show the highest interfacial hole concentration under TG bias, followed by OFF bias, whereas ON bias produces the lowest concentration. The mechanism is governed by the bias-dependent electric field during irradiation. With no applied bias (NO), holes diffuse outward from the central region. Under OFF bias, the drain voltage counteracts part of this natural outward redistribution and skews the charge profile. Under TG bias, the combined source/drain potentials oppose outward diffusion from both sides and concentrate holes near the middle of the channel. Under ON bias, the applied field enhances outward transport and therefore lowers the hole concentration in the central region.

The close agreement between the simulated and measured degradation trends supports the physical picture of TID degradation in FDSOI MOSFETs: bias-dependent threshold-voltage shifts, electric-field-driven hole transport and trapping, and a direct relationship between trapped charge and accumulated dose. These validated simulation results provide the physics-based dataset required for the neural-network framework described in Section 4.

## 4. Neural Network-Based Reliability Analysis Methodology

### 4.1. Radiation-Induced Fixed Charge Simulations for Neural Network Training

Although the full Radiation model can reproduce the TID response and bias-dependent hole distribution in the BOX, its computational cost limits large-scale data generation. In the source study, a Radiation-model scan requires approximately 20 times longer than the corresponding Fixcharge-model calculation, whereas the Fixcharge approach produces substantially more data points within the same computational budget. Coupling the Fixcharge model with neural-network training therefore preserves the relevant degradation trend while enabling a larger training dataset and faster DNN convergence. The Fixcharge model is used for the initial large-scale generation of training data, and the Radiation model is retained for validation subsets.

**Figure 5:**
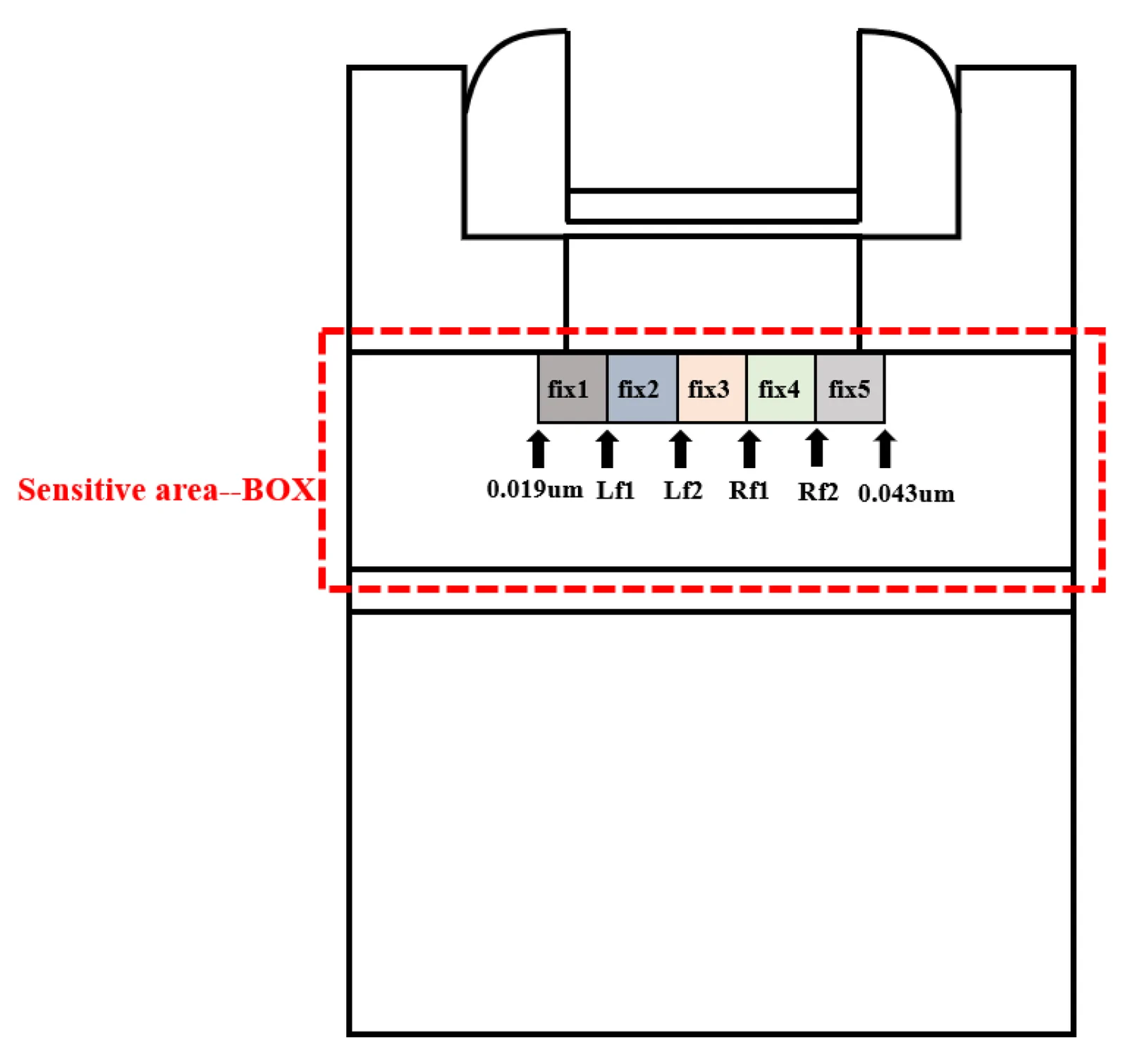
Schematic of the regional Fixcharge model used to represent the radiation-sensitive BOX region.

The proposed Fixcharge model is illustrated in Figure 5. The BOX region near the critical BOX/channel interface is divided into five subregions (fix1–fix5). The outer boundaries of fix1 and fix5 are fixed at x = 0.019 μm and x = 0.043 μm, respectively, while the internal boundaries Lf1, Lf2, Rf1, and Rf2 are adjustable. Each region has a uniform height of 5 nm. A fixed-charge concentration (conc1–conc5) is assigned to each region to approximate the radiation-induced hole distribution obtained from the Radiation model.

**Figure 6:**
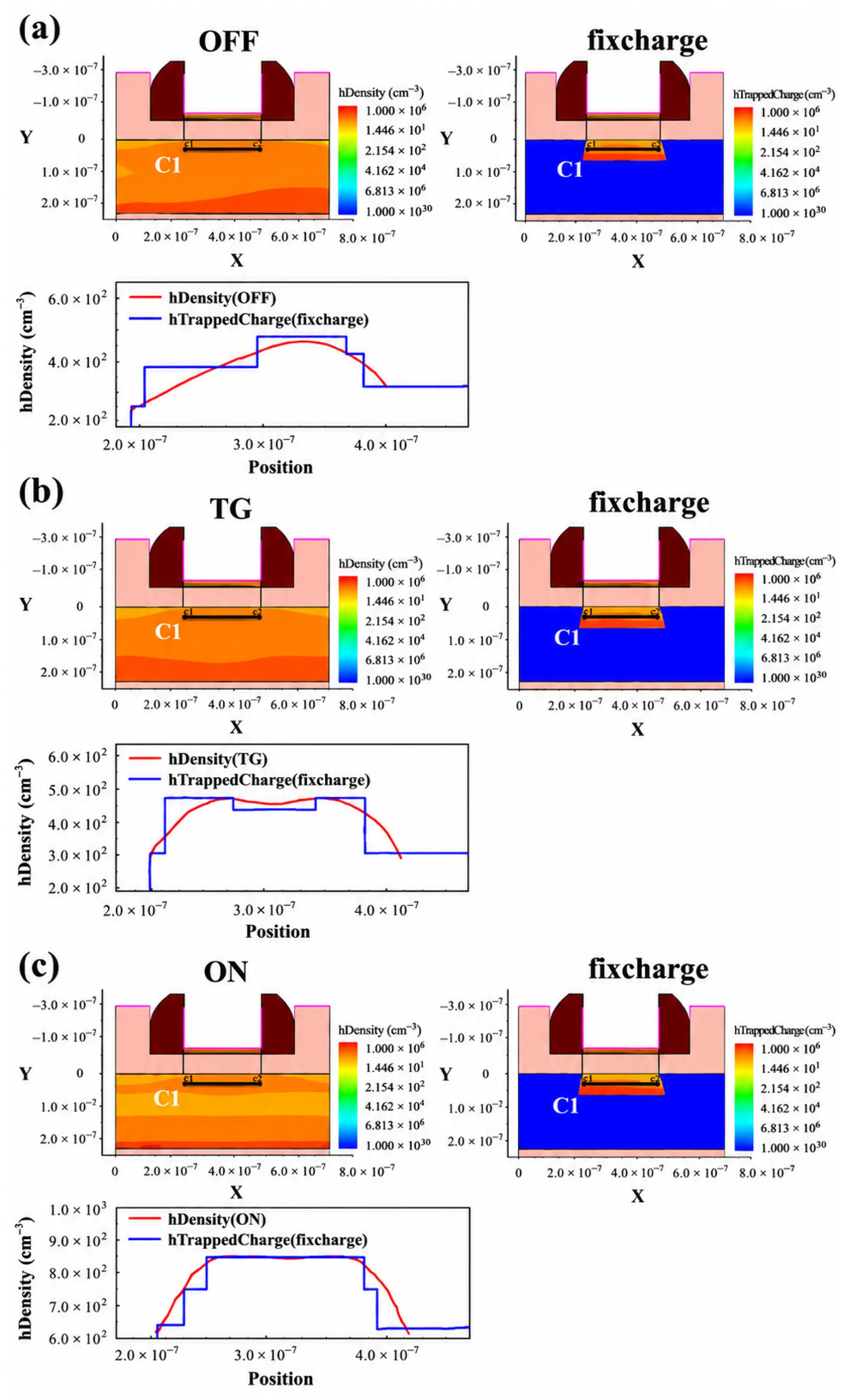
Comparison of Radiation-model and regional Fixcharge-model hole distributions in the BOX under (**a**) OFF, (**b**) TG, and (**c**) ON bias conditions.

Parameter calibration is performed using the 1500 s irradiation results corresponding to the 0–250 krad(Si) dose interval. Representative calibrated parameters for the OFF, TG, and ON bias conditions are listed in Table 4, Table 5 and Table 6. To validate the accuracy of the Fixcharge model, we compared the hole density distributions along the C1 interface in the buried oxide layer against the Radiation model simulation results, as shown in Figure 6.

The regional Fixcharge model reproduces the Radiation-model charge distribution with high consistency at the BOX/channel interface under OFF, TG, and ON biases. This agreement verifies that the simplified representation captures the spatially nonuniform trapped-charge patterns relevant to the TID degradation mechanism while providing a much more efficient data-generation route.

### 4.2. A Neural Network-Based Analysis of Total Ionizing Dose Effects in FDSOI Devices

**Figure 7:**
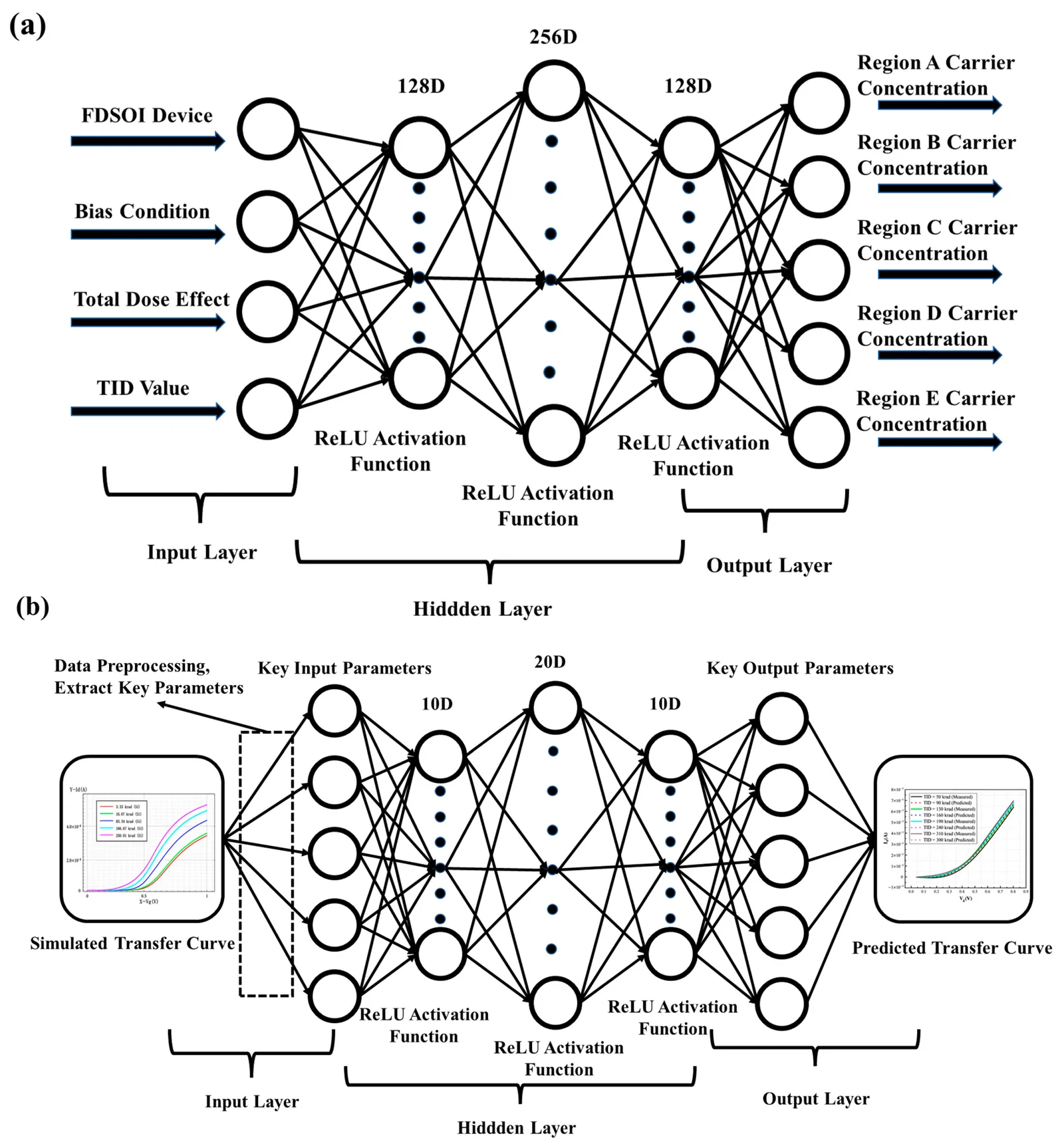
Deep-learning framework for TID analysis: (**a**) network for predicting the BOX hole-concentration distribution; (**b**) network for predicting/correcting the FDSOI transfer characteristics.

To combine the physical fidelity of the Radiation model with the data-generation efficiency of the Fixcharge model, a two-stage deep-neural-network framework is constructed for TID analysis of FDSOI MOSFETs. The DNN models were implemented in Python 3.8 using PyTorch version 2.2.2. Scikit-learn version 1.3.2 was employed for data preprocessing and feature standardization. Two fully connected neural networks were constructed for the TID reliability analysis. The first-stage network maps the irradiation conditions to the charge distribution in the five sensitive BOX regions using a 4–128–256–128–5 architecture with ReLU activation, whereas the second-stage network employs a 5–20–10–5 architecture to map the TCAD-derived transfer-curve features to the corresponding corrected output features. The complete dataset was divided into training, validation, and independent test subsets with a ratio of 70:15:15. To avoid information leakage between highly correlated neighboring data points, the partition was performed at the irradiation-condition level rather than by randomly splitting individual points. All samples corresponding to a given dose/bias condition were assigned exclusively to one subset. The first network maps device type, TID information, irradiation bias, and total dose to the hole concentrations in the five BOX-sensitive regions. The predicted regional charge distribution is then applied to the Fixcharge model to rapidly generate the transfer characteristics. A second network corrects the simulated transfer curve to account for the residual discrepancy between TCAD and experiment. The overall framework is shown in Figure 7.

Hyperparameter selection was performed using the validation subset. The hidden-layer widths were determined through repeated trials by considering both validation error and model complexity. For the first-stage network, an expanding–compressing structure was examined, and the 128–256–128 configuration was finally selected because increasing the feature dimension improved the representation of the nonlinear dose–bias–charge relationship, whereas the subsequent compression reduced redundant features and model complexity. The second-stage network was similarly reduced to a 20–10 hidden-layer structure because its input and output dimensions were substantially smaller. Adam was used for optimization with an initial learning rate of 0.001, and the networks were trained for up to 3000 epochs using a batch size of 32. L2 regularization was applied with a weight-decay coefficient of 0.00155, selected according to validation performance. In addition, a dynamically decreasing dropout strategy was employed, with an initial dropout probability of 0.3002, a decay step of 0.0372, and a decay cycle of 500 epochs. The model checkpoint giving the lowest validation loss was retained for independent testing.

**Figure 8:**
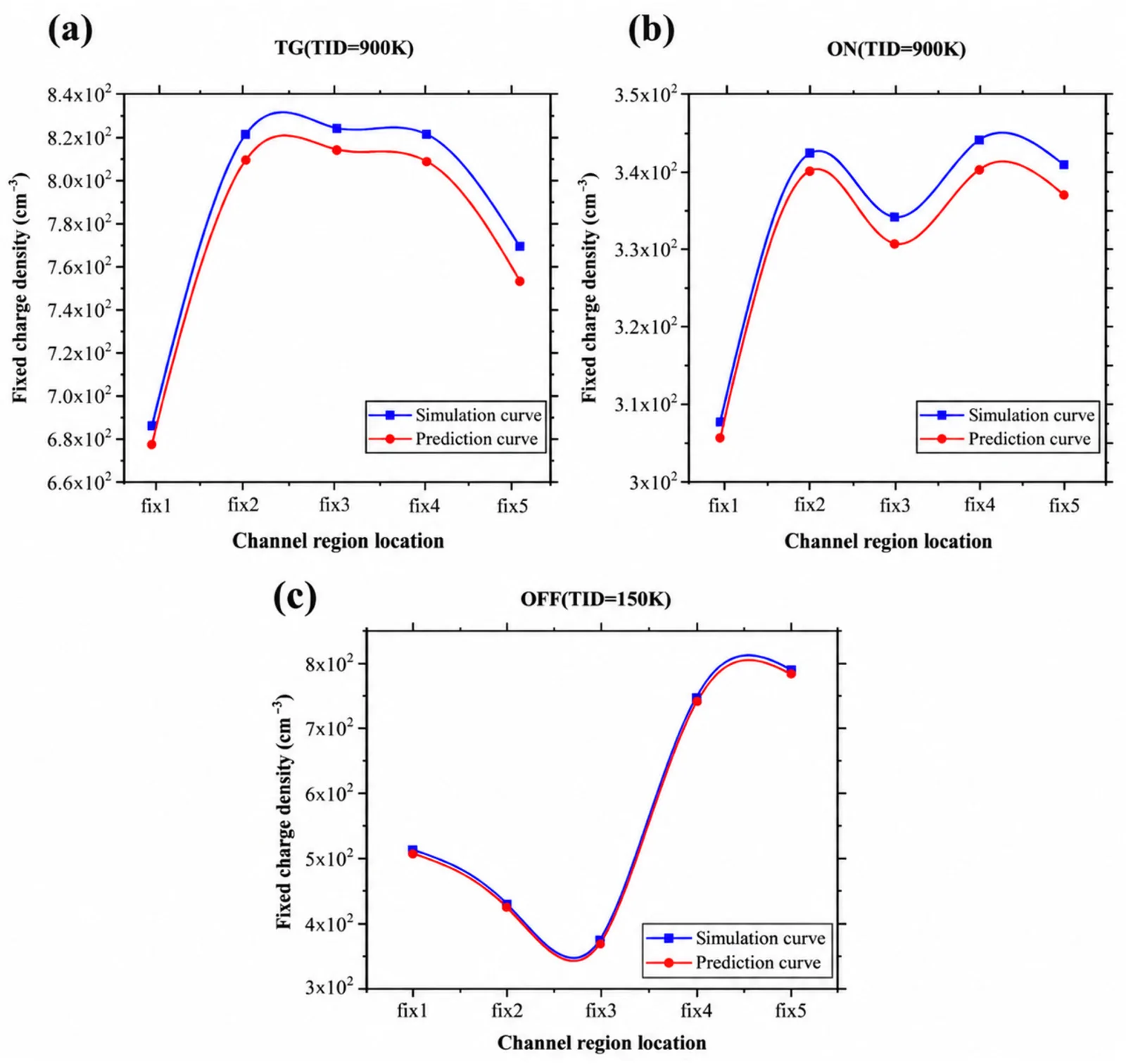
Predicted fixed-charge distributions under (**a**) TG bias, (**b**) ON bias, and (**c**) OFF bias compared with Radiation-model results.

For the first-stage network, the five sensitive BOX regions define the output vector. A fully connected 128–256–128 hidden-layer architecture with ReLU activation is used to learn the nonlinear relationship among total dose, irradiation bias, and spatial charge distribution. The model was evaluated under representative TG, ON, and OFF irradiation conditions. To evaluate the predictive capability of the DNN over a broader dose range, both in-range and out-of-range cases were considered. The 150 krad(Si) case lies within the experimentally validated dose range, whereas the 900 krad(Si) cases represent extrapolation beyond the available experimental range. In the latter cases, the experimentally validated Radiation-model TCAD results were used as the physics-based reference rather than experimental measurements. As shown in Figure 8, the predicted distributions closely follow the Radiation-model results for all three representative bias conditions. At TG bias and 900 krad(Si), RMSE = 0.122 and MAE = 0.120; at ON bias and 900 krad(Si), RMSE = 0.037 and MAE = 0.034; and at OFF bias and 150 krad(Si), RMSE = 0.034 and MAE = 0.032. Using the relative prediction accuracy defined below, the average agreement between the predicted and Radiation-model fixed-charge concentrations over the five BOX-sensitive regions exceeds 95% for all three representative cases.

The RMSE and MAE are calculated as follows:

(1) $$\mathrm{RMSE}=\sqrt{\frac{1}{n}\sum_{i=1}^{n}{\left(\mathrm{yi}-\hat{\mathrm{yi}}\right)}^{2}}$$

(2) $$\mathrm{MAE}=\frac{1}{n}\sum_{i=1}^{n}\left|\mathrm{yi}-\hat{\mathrm{yi}}\right|$$

where *n* is the number of observations, *yi* is the reference value, and *ŷi* is the corresponding model prediction. Lower RMSE and MAE values indicate better agreement between the prediction and the reference data.

**Figure 9:**
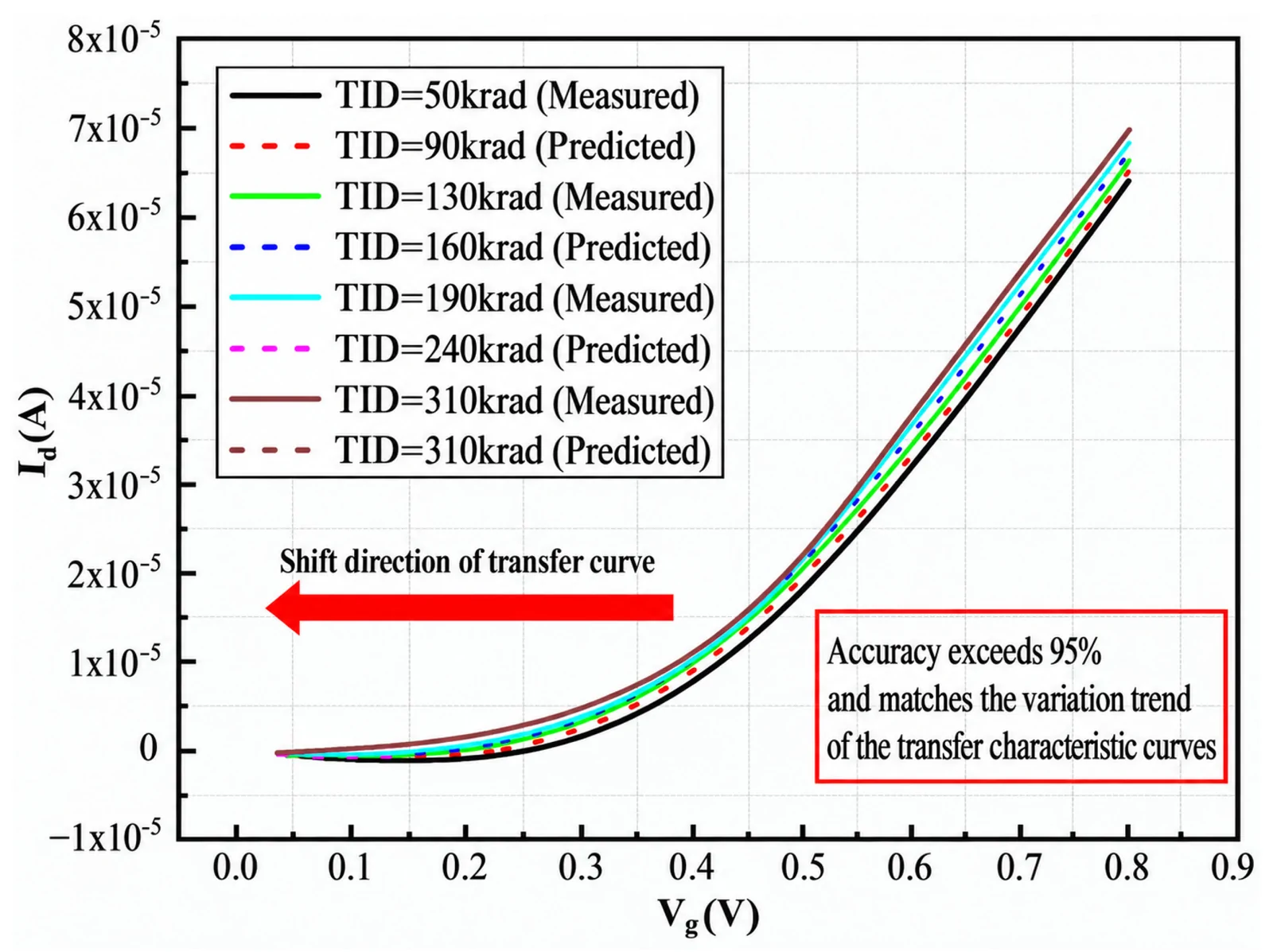
Comparison of measured and predicted transfer characteristics at different total-ionizing-dose levels.

In addition to RMSE and MAE, the relative prediction accuracy was used to quantify the agreement between the DNN predictions and the corresponding reference values. For each data point, the relative prediction accuracy was calculated as:

(3) $$A_{i}=(1-\frac{\left|\hat{\mathrm{yi}}-\mathrm{yi}\right|}{\left|\mathrm{yi}\right|})\times 100\%$$

and the average prediction accuracy over n evaluated points was calculated as:

(4) $$A_{\mathrm{avg}}=\frac{1}{n}\sum_{i=1}^{n}A_{i}=[1-\frac{1}{n}\sum_{i=1}^{n}\frac{\left|\hat{\mathrm{yi}}-\mathrm{yi}\right|}{\left|\mathrm{yi}\right|}]\times 100\%$$

where *yi* denotes the corresponding reference value and *ŷi* denotes the DNN-predicted value. For the BOX-charge prediction shown in Figure 8, the calculation was performed by comparing the predicted and Radiation-model fixed-charge concentrations in each of the five sensitive BOX regions. The relative prediction accuracy was calculated for the five regions under each evaluated irradiation condition, and the resulting average accuracy exceeded 95% for the representative cases. For the transfer-characteristic prediction shown in Figure 9, the same relative-comparison criterion was applied to the predicted and reference current values at the selected gate-voltage points for each dose condition. The evaluated transfer characteristics likewise exhibited an average relative prediction accuracy above 95%.

After the five BOX-region concentrations are obtained, the Fixcharge model rapidly generates the corresponding FDSOI MOSFET transfer characteristic. Because simplified geometry and approximate physical parameters can still produce deviations between TCAD and measurement, a second data-driven network is used to correct the transfer curve. To address the different samp ling densities of the simulated and measured curves, five key current points at fixed gate voltages are extracted to represent the turn-on and conduction behavior. A fully connected 5–20–10–5 network maps the simulated key points to their experimental counterparts. Input and output data are standardized before training; ReLU is used in the hidden layers, mean-square error is used as the loss function, and Adam is used for parameter optimization.

To mitigate overfitting, L2 regularization and dynamic dropout were jointly employed. L2 regularization constrains excessive weight growth, whereas dynamic dropout reduces neuron co-adaptation. Their combination improves model generalization and robustness under previously unseen irradiation conditions.

Figure 9 compares the DNN-predicted transfer characteristics with the corresponding TCAD-generated reference characteristics. The TCAD model was first calibrated and validated against experimental measurements within the experimentally investigated dose range of 0–250 krad(Si). After this validation, the model was used to generate reference data at higher dose levels. At 310 krad(Si), which is slightly beyond the experimental dose range, the DNN-predicted transfer characteristic closely agrees with the TCAD reference curve, with an overall reported accuracy above 95%. Across multiple dose levels, both the predicted and measured transfer curves progressively shift toward negative gate voltage as TID increases, reproducing the threshold-voltage reduction associated with radiation-induced positive-charge accumulation. Thus, the framework captures not only a single-dose transfer characteristic but also the dose-dependent electrical degradation trend.

In summary, the first DNN learns the nonlinear mapping from total dose and irradiation bias to the hole concentrations in five sensitive BOX regions, while the second DNN maps TCAD-derived key points to experimental transfer-characteristic key points. The two networks preserve the bias-dependent spatial charge patterns and the dose-dependent negative shift in the transfer characteristics, enabling rapid reliability analysis with a reported average prediction accuracy above 95% for the evaluated cases.

## 5. Conclusions

TID effects in 22 nm FDSOI devices were investigated using X-ray irradiation measurements, a calibrated TCAD model, a regional fixed-charge representation, and a two-stage DNN framework. The TCAD analysis shows that radiation-induced holes accumulate in the BOX and near the BOX/top-silicon interface, where the resulting positive fixed charge modulates the channel potential. With increasing dose, the threshold voltage decreases, the transfer characteristics shift toward negative gate voltage, and the off-state behavior and electrical reliability degrade. Irradiation bias changes the internal electric-field distribution and therefore modifies the direction of hole transport, the location of charge accumulation, and the degree of device degradation.

To address the high computational cost of the conventional Radiation model, the radiation-sensitive BOX region was divided into five subregions and represented by a nonuniform regional fixed-charge model. This simplified model remains consistent with the Radiation model in terms of both charge-distribution and transfer-characteristic trends while reducing the average computation time by approximately a factor of 20, thereby providing an efficient route for generating DNN training data.

On this basis, a two-stage DNN is used to link irradiation conditions to device degradation. The first stage predicts the charge concentrations in the five BOX regions from total dose and irradiation bias; the second stage predicts and corrects the transfer characteristics using the simulated current features. The framework reproduces the dose-dependent threshold-voltage decrease and negative transfer-curve shift with an average reported prediction accuracy above 95%, combining physics-based fidelity with data-driven computational efficiency for TID reliability assessment and radiation-aware design of FDSOI devices.

Nevertheless, an important limitation of the present study should be acknowledged. Although irradiated and unirradiated devices were used for comparison, the irradiation measurements were obtained from a single 22 nm FDSOI device. Therefore, the reported results describe the dose-dependent evolution of this individual device, and device-to-device variability and experimental repeatability across multiple irradiated devices have not yet been statistically evaluated. At this stage, the primary objective of the proposed framework is to demonstrate the feasibility of applying neural-network-based surrogate modeling to semiconductor radiation-effect analysis. Future work will extend the irradiation experiments to a larger number of FDSOI devices and a broader range of irradiation conditions, thereby providing a substantially enlarged dataset to further develop the machine-learning model and improve its statistical robustness, generalization capability, and reliability.

## Figures and Tables

**Table 1:** DC parameters of the FDSOI MOSFET under X-ray irradiation.

Total Dose/krad(Si)	0	25	75	150	250
***I_on_*** **/μA μm^−1^**	814	824	842	862	883
***I_off_*** **/nA μm^−1^**	120	144	224	340	582
***I_on_*** **/*I_off_***	6.80 × 10^3^	5.72 × 10^3^	3.76 × 10^3^	2.53 × 10^3^	1.52 × 10^3^
***V_th_*** **/V**	0.288	0.282	0.267	0.256	0.233

**Table 2:** Key parameters used for device-model calibration.

Symbol	Parameter	Value
LG	Gate length	24 nm
Tchannel	Channel thickness	7 nm
TBOX	BOX thickness	20 nm
TUB	Back-gate layer thickness	5 nm
TSUB	Substrate thickness	45 nm
NSD	Source/drain doping	1.3 × 10^19^ cm^−3^
NUB	Back-gate doping	1 × 10^18^ cm^−3^
NSUB	Substrate doping	1 × 10^18^ cm^−3^
NCH	Channel doping	1 × 10^14^ cm^−3^
WK	Gate work function	4.65 eV

**Table 3:** Bias conditions used for the total-ionizing-dose irradiation simulations.

Bias Condition	Gate	Source	Substrate
OFF	0 V	0 V	0.7 V
TG	0 V	0.7 V	0.7 V
ON	0.7 V	0 V	0 V

**Table 4:** Fixcharge-model parameters for the OFF-bias condition.

Symbol	Value	Symbol	Value
Lf1	0.021 μm	conc1	2.28 × 10^2^ cm^−3^
Lf2	0.031 μm	conc2	3.89 × 10^2^ cm^−3^
Rf1	0.039 μm	conc3	4.92 × 10^2^ cm^−3^
Rf2	0.041 μm	conc4	4.29 × 10^2^ cm^−3^
		conc5	3.08 × 10^2^ cm^−3^

**Table 5:** Fixcharge-model parameters for the TG-bias condition.

Symbol	Value	Symbol	Value
Lf1	0.021 μm	conc1	2.68 × 10^2^ cm^−3^
Lf2	0.027 μm	conc2	4.42 × 10^2^ cm^−3^
Rf1	0.035 μm	conc3	4.18 × 10^2^ cm^−3^
Rf2	0.041 μm	conc4	4.43 × 10^2^ cm^−3^
		conc5	2.47 × 10^2^ cm^−3^

**Table 6:** Fixcharge-model parameters for the ON-bias condition.

Symbol	Value	Symbol	Value
Lf1	0.021 μm	conc1	6.37 × 10^2^ cm^−3^
Lf2	0.023 μm	conc2	7.63 × 10^2^ cm^−3^
Rf1	0.038 μm	conc3	8.79 × 10^2^ cm^−3^
Rf2	0.041 μm	conc4	7.61 × 10^2^ cm^−3^
		conc5	6.13 × 10^2^ cm^−3^

## Data Availability

The data that support the findings of this study are available from the corresponding author upon reasonable request.
